# Wnt2 complements Wnt/β-catenin signaling in colorectal cancer

**DOI:** 10.18632/oncotarget.6133

**Published:** 2015-10-15

**Authors:** Youn-Sang Jung, Sohee Jun, Sun Hye Lee, Amrish Sharma, Jae-Il Park

**Affiliations:** ^1^ Department of Experimental Radiation Oncology, The University of Texas MD Anderson Cancer Center, Houston, TX, USA; ^2^ Program in Genes and Development, The University of Texas MD Anderson Cancer Center, Houston, TX, USA; ^3^ Graduate School of Biomedical Sciences at Houston, The University of Texas Health Science Center and MD Anderson Cancer Center, Houston, TX, USA

**Keywords:** Wnt, β-catenin, Wnt2, colorectal cancer

## Abstract

Wnt2 is implicated in various human cancers. However, it remains unknown how Wnt2 is upregulated in human cancer and contributes to tumorigenesis. Here we found that Wnt2 is highly expressed in colorectal cancer (CRC) cells. In addition to co-expression of Wnt2 with Wnt/β-catenin target genes in CRC, knockdown or knockout of Wnt2 significantly downregulates Wnt/β-catenin target gene expression in CRC cells. Importantly, depletion or ablation of endogenous Wnt2 inhibits CRC cell proliferation. Similarly, neutralizing secreted Wnt2 reduces Wnt target gene expression and suppresses CRC cell proliferation. Conversely, Wnt2 increases cell proliferation of intestinal epithelial cells. Intriguingly, *WNT2* expression is transcriptionally silenced by EZH2-mediated H3K27me3 histone modification in non-CRC cells, However, *WNT2* expression is de-repressed by the loss of PRC2's promoter occupancy in CRC cells. Our results reveal the unexpected roles of Wnt2 in complementing Wnt/β-catenin signaling for CRC cell proliferation.

## INTRODUCTION

Wnt signaling is essential for stem cell regulation in development and tissue homeostasis [1]. Wnt signaling plays key roles in cell proliferation, cell fate determination, cell migration, and cell polarity during embryonic development and stem cell regulation [2]. Wnt ligands bind to Frizzled receptors and low-density lipoprotein receptor-related protein 5/6 co-receptors, which stabilizes β-catenin protein by inhibiting the protein destruction complex composed of adenomatous polyposis coli, Axin, casein kinase 1, and glycogen synthase kinase 3. Subsequently, stabilized β-catenin protein is translocated into the nucleus and replaces T cell factor (TCF)–associated corepressors with coactivators, which results in the transcriptional activation of the β-catenin target genes [2]. However, deregulated Wnt signaling leads to intestinal tumorigenesis. Frequent genetic mutations in Wnt signaling components have been closely associated with human colorectal cancer (CRC) [3]. In mouse models, genetic mutations that lead to the hyperactivation of Wnt signaling induce mammary tumors and intestinal adenomas [6], recapitulating the pivotal roles of Wnt signaling in intestinal tumor initiation. Such hyperactivation of Wnt/β-catenin signaling is driven by genetic mutations in core components of Wnt signaling. For example, more than 70% of CRC cells display somatic mutation in Wnt signaling components including *APC*, *β-catenin/CTNNB1*, or *AXIN1* [4, 5]. Additionally, it was shown that transcriptional silencing of Wnt signaling antagonists induces Wnt signaling hyperactivation in CRC cells [7-13]. Moreover, it was suggested that high expression of Wnt3A is associated with intestinal tumorigenesis [14]. Among 19 Wnt ligands in mammals, Wnt1, Wnt3A, Wnt8, and Wnt10 transduce Wnt signaling via β-catenin, called as canonical Wnt ligands [15]. Wnt5A and Wnt11 activate small GTPases (RhoA and Rac1), Ca^2+^ signaling, protein kinase C, or planar cell polarity, called as non-canonical Wnt signal transduction [16]. Due to diverse impacts of 19 Wnt ligands to cellular functions in combination with 10 Frizzled receptors, the effects of Wnt ligands on tumorigenesis still remains ambiguous.

Wnt2, a member of the *WNT* gene family, directs cell specification during development [17]. Wnt2 plays a critical role in development. In mouse models, genetic ablation of *WNT2* induces vascular defects [18]. In Drosophila, Wnt2 is required for development of male reproductive tract [19]. Intriguingly, Wnt2 upregulation was observed in various human cancers [20-23]. It was shown that Wnt2 plays tumorigenic roles in several cancers including non-small-cell lung cancer, pancreatic cancer, ovary cancer, esophageal cancer, and gastro-intestinal cancer [24-29]. Also, it was suggested that upregulation of Wnt2 is likely to be an early event during intestinal tumorigenesis [21]. In cancer cells, Wnt2 expression is associated with anchorage-independent cell survival, metastasis, and tumor invasion [24, 30]. Moreover, the expression of Wnt2 is implicated in activating/stabilizing β-catenin, similar to other canonical (β-catenin-mediated) Wnt ligands [27, 31-35]. And, the blockade of Wnt2 destabilizes β-catenin protein in CRC cells [36]. It was shown that Wnt2 is enriched in circulating pancreatic cancer cells [24]. Despite significant implication of Wnt2 in malignant cancer, it remains unclear how Wnt2 contributes to tumorigenesis.

Herein, we identified Wnt2 as a key ligand that complements Wnt/β-catenin signaling activity in CRC. To understand the pathologic impacts of Wnt ligands to intestinal tumorigenesis, we analyzed the expression of 19 Wnt ligands in CRC cells, and found that Wnt2 is significantly upregulated in CRC and hyperactivates β-catenin. Depletion of endogenous Wnt2 inhibits CRC cell proliferation, accompanied with the decreased Wnt/β-catenin signaling activity. We also found that Polycomb Repressive Complex 2 (PRC2) epigenetically controls expression of *WNT2*. Our results suggest that Wnt2 enhances Wnt/β-catenin signaling in an autocrine manner, which contributes to intestinal tumorigenesis.

## RESULTS

### Expression of Wnt2 in CRC

**Figure 1:**
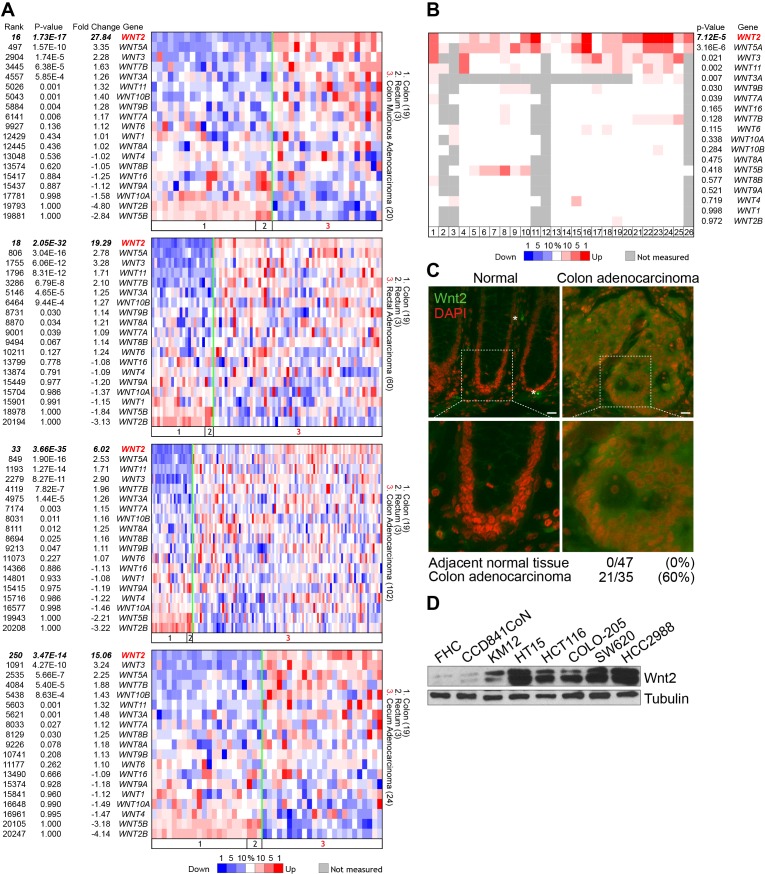
Expression of Wnt2 in CRC **A.** Oncomine analysis of *WNT* ligands in CRC. P value < 0.0001; fold change > 2. TCGA CRC datasets. **B.** Oncomine analysis of *WNT2* expression in CRC datasets. P value < 0.0001; fold change > 2. Dataset information: 1. Rectal Adenocarcinoma vs. Normal / Gaedcke Colorectal, Genes Chromosomes Cancer, 2010; 2. Colorectal Adenoma Epithelia vs. Normal / Gaspar Colon, Am J Pathol, 2008; 3. Colorectal Carcinoma vs. Normal / Graudens Colon, Genome Biol, 2006; 4. Colorectal Carcinoma vs. Normal / Hong Colorectal, Clin Exp Metastasis, 2010; 5. Cecum Adenocarcinoma vs. Normal / Kaiser Colon, Genome Biol, 2007; 6. Colon Adenocarcinoma vs. Normal / Kaiser Colon, Genome Biol, 2007; 7. Colon Mucinous Adenocarcinoma vs. Normal / Kaiser Colon, Genome Biol, 2007; 8. Rectal Adenocarcinoma vs. Normal / Kaiser Colon, Genome Biol, 2007; 9. Rectal Mucinous Adenocarcinoma vs. Normal / Kaiser Colon, Genome Biol, 2007; 10. Rectosigmoid Adenocarcinoma vs. Normal / Kaiser Colon, Genome Biol, 2007; 11. Colon Adenocarcinoma vs. Normal / Ki Colon, Int J Cancer, 2007; 12. Colon Adenocarcinoma vs. Normal / Notterman Colon, Cancer Res, 2001; 13. Colon Adenoma vs. Normal / Sabates-Bellver Colon, Mol Cancer Res, 2007; 14. Rectal Adenoma vs. Normal / Sabates-Bellver Colon, Mol Cancer Res, 2007; 15. Colorectal Adenocarcinoma vs. Normal / Skrzypczak Colorectal, PLoS One, 2010; 16. Colorectal Carcinoma vs. Normal / Skrzypczak Colorectal, PLoS One, 2010; 17. Colon Adenoma Epithelia vs. Normal / Skrzypczak Colorectal 2, PLoS One, 2010; 18. Colon Adenoma vs. Normal / Skrzypczak Colorectal 2, PLoS One, 2010; 19. Colon Carcinoma Epithelia vs. Normal / Skrzypczak Colorectal 2, PLoS One, 2010; 20. Colon Carcinoma vs. Normal / Skrzypczak Colorectal 2, PLoS One, 2010; 21. Cecum Adenocarcinoma vs. Normal / TCGA Colorectal, Nature, 2012; 22. Colon Adenocarcinoma vs. Normal / TCGA Colorectal, Nature, 2012; 23. Colon Mucinous Adenocarcinoma vs. Normal / TCGA Colorectal, Nature, 2012; 24. Rectal Adenocarcinoma vs. Normal / TCGA Colorectal, Nature, 2012; 25. Rectal Mucinous Adenocarcinoma vs. Normal / TCGA Colorectal, Nature, 2012; 26. Colon Carcinoma vs. Normal / Zou Colon, Oncogene, 2002. **C.** Wnt2 expression in human CRC tissues. Human CRC tissue microarray was analyzed for Wnt2 expression by immunofluorescent staining. 4′,6-diamidino-2-phenylindole, dihydrochloride (DAPI): nuclear counterstaining. **D.** Expression of Wnt2 protein in non-CRC (FHC and CCD841CoN) and CRC cell lines. IB analysis.

We initially sought to investigate the impacts of each Wnt ligand to intestinal tumorigenesis. To do this, we assessed expression of Wnt ligands in CRC cells. We performed in silico expression analysis of Wnt ligands in CRC cells, using publicly available database (www.oncomine.org). We found that, among 19 Wnt ligands, *WNT2* and *WNT5A* are highly upregulated in colon mucinous adenocarcinoma, rectal adenocarcinoma, colon adenocarcinoma, and cecum adenocarcinoma (Figure 1A). Additionally, analysis of 26 independent datasets showed that expression of *WNT2* and *WNT5A* is highly elevated in CRC, while underexpressed or not expressed in normal intestine (Figure 1B). These results were further validated by immunostaining of CRC tissue microarray for *Wnt2* expression. We observed that unlike normal colorectum tissues, 60% (21/35) of human CRC tissues expressed high level of Wnt2 (Figure 1C). Consistently, immunoblot (IB) analyses confirmed that Wnt2 is highly expressed in CRC cell lines (Figure 1D). These results suggest that Wnt2 expression is significantly upregulated in CRC.

### Wnt2 enhances Wnt/β-catenin signaling activity in CRC

**Figure 2:**
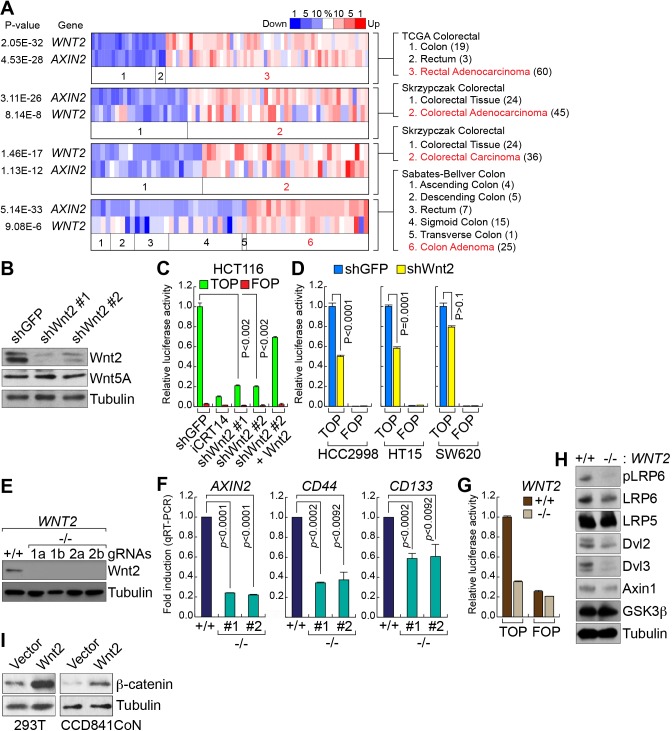
*Wnt2* is required for β-catenin target gene activation in CRC cells **A.** Co-expression of *WNT2* with *AXIN2* in CRC. Co-expression analysis of *WNT2* with *AXIN2*, using Oncomine database. P value < 0.0001; fold change > 2. **B.** Depletion of endogenous Wnt2 by shRNAs. Lentiviruses encoding two different shRNAs (#1 and #2) were stably transduced into HCT116 cells. IB assays. Of note, Wnt5A expression was not affected by Wnt2 depletion. **C.** and **D.** Downregulation of β-catenin transcriptional activity by *Wnt2* KD in CRC cells. HCT116 cells (shGFP or shWnt2) were transiently transfected with pMegaTOPFLASH (TOP) or pMegaFOPFLASH (FOP) β-catenin luciferase reporter plasmids, with pRenilla plasmids (internal control). 48 hours after transfection, cells were analyzed for luciferase activity **C.**. Other CRC cell lines **D.**. iCRT14 served as a positive control for inhibition of β-catenin activity. Wnt2-expressing plasmids were co-transfected for rescue assays. **E.** KO of *WNT2* alleles by CRISPR gene targeting system. HCT116 cells were transduced with lentiviruses encoding Cas9 and gRNAs (1 and 2 indicate two different gRNAs). IB analysis. **F.** Downregulation of β-catenin target genes by *WNT2* KO. HCT116 (*WNT2* WT vs. *WNT2* KO) cells were analyzed by quantitative reverse transcriptase PCR (qRT-PCR) of *AXIN2*, *CD44*, and *CD133*. **G.** Suppression of β-catenin transcriptional activity by *WNT2* KO. β-catenin reporter assays of HCT116 (*WNT2* WT vs. *WNT2* KO). **H.** Dephosphorylation of LRP6 by *WNT2* KO. IB analysis of HCT116 (*WNT2* WT vs. *WNT2* KO). **I.** Upregulation of β-catenin protein by Wnt2. 293T and CCD841CoN cells were transfected with plasmids encoding Wnt2. 24 hours after transfection, cells were analyzed for IB of β-catenin.

While Wnt5A transduces non-canonical (not mediated by β-catenin) Wnt signaling [37], Wnt2 activates canonical Wnt signaling and upregulates β-catenin target genes [34, 35, 38, 39]. This led us to test whether Wnt2 is required for full activation of Wnt/β-catenin signaling activity in CRC cells. Thus, we examined the correlation between *WNT2* and Wnt signaling activation by assessing the co-expression of *WNT2* and *AXIN2*, a well-established β-catenin target gene [40, 41]. Analyses of Oncomine datasets showed that *WNT2* expression is strongly co-related with expression of *AXIN2*, compared with other Wnt ligands (Figure 2A and Supplementary Figure 1), implying that Wnt2 might be associated with Wnt signaling in CRC cells.

Next, to examine the effect of Wnt2 on Wnt/β-catenin signaling in CRC, we employed the loss-of-function approaches by transducing lentiviruses encoding short-hairpin RNA (shRNA) against green fluorescence (GFP; shGFP) or Wnt2 (shWnt2) (Figure 2B), as previously performed [42, 43]. We found that Wnt2 knockdown (KD) markedly downregulates β-catenin reporter activity (TOPFLASH luciferase) in HCT116 cells, which was rescued by ectopic expression of Wnt2 (Figure 2C). iCRT14, an inhibitor for β-catenin:TCFs binding, served as a positive control. Additionally, other CRC cell lines (HCC2998, HT15, and SW620) similarly displayed the downregulation of β-catenin reporter activity by endogenous Wnt2 KD (Figure 2D). To better confirm the effects of Wnt2 depletion on Wnt signaling, we employed the clustered regularly interspaced short palindromic repeat (CRISPR) gene targeting system using lentivirus encoding Cas9 and guide RNA (gRNA). IB assays showed that two different gRNAs successfully target *WNT2* alleles (Figure 2E), which was also validated by genomic DNA sequencing (data not shown). Similar to the effects of Wnt2 KD, *WNT2* knockout (KO) cells displayed significantly reduced expression of β-catenin target genes (*AXIN2*, *CD44*, and *CD133*) (Figure 2F) and β-catenin reporter activity (Figure 2G), compared to *WNT2* wild-type (WT) cells. We also analyzed the effects of *WNT2* KO on activity of each Wnt signaling component. We found that *WNT2* KO cells exhibited decreased phosphorylation of LRP6, compared to *WNT2* WT cells (Figure 2H). Next, we tested whether Wnt2 activates β-catenin in non-CRC cells. Indeed, ectopic expression of Wnt2 upregulates β-catenin protein in 293T and CCD841CoN intestinal epithelial cells (IECs) (Figure 2I). These results suggest that Wnt2 is required for maintenance of Wnt signaling activity in CRC cells.

### Wnt2 is required for CRC cell proliferation

**Figure 3:**
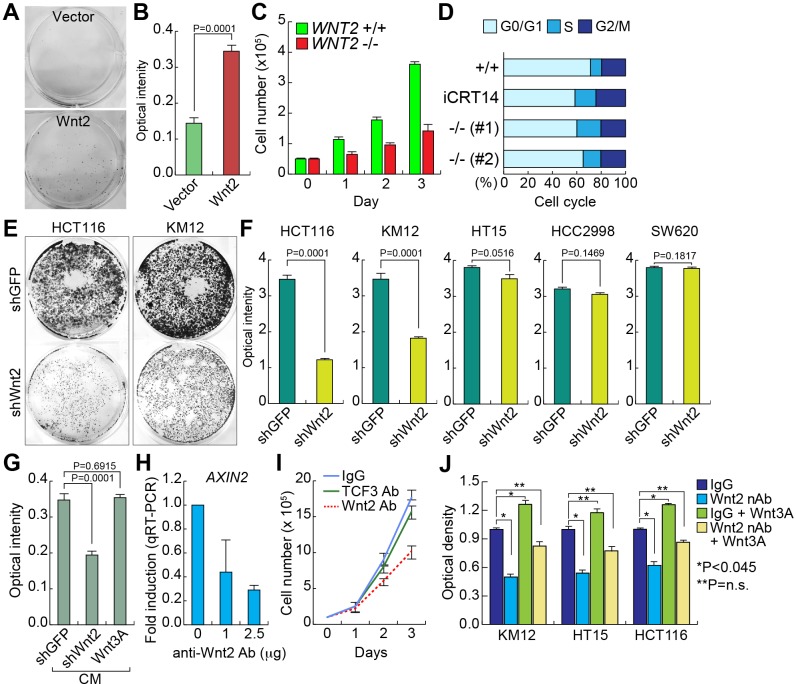
Wnt2 is required for CRC cell proliferation **A.** and **B.** Increased cell proliferation by Wnt2. CCD841CoN IECs were stably transfected with Wnt2-expressing plasmid. Then, the equal number of cells were plated and cultured for 20 days. Crystal violet staining **A.**; quantification of optical intensity of crystal violet stained cells **B.**. **C.** Decreased cell proliferation by *WNT2* KO. The equal number of HCT116 (*WNT2* WT vs. *WNT2* KO) cells were plated and counted in a different time course. **D.** Cell cycle analysis of *WNT2* KO cells. HCT116 cells were analyzed using flow cytometry. **E.** and **F.** Depletion of Wnt2 reduced CRC cell proliferation. CRC cells were stably transduced with lentivirus encoding shRNAs (shGFP: control; shWnt2). Then, the equal number of cells were plated and grown for 14 days for crystal violet staining **E.** and quantification **F.**. **G.** Decreased cell proliferation by Wnt2 KD CM. KM12 cells were transfected with plasmids (empty vector: control; shWnt2; Wnt3A). After transfection, KM12 cells were incubated with serum free DMEM for collection of CM. Then, each CM was treated to HCT116 cells. Cell proliferation (crystal violet staining quantification) was monitored after were monitored for 20 days of CM treatment. **H.** Downregulation of *AXIN2* by neutralizing Wnt2 protein. HCT116 cells were treated with anti-Wnt2 antibody. 24 hours after treatment, cells were analyzed for qRT-PCR of *AXIN2*. HPRT served as an internal control. **I.** Inhibition of cell proliferation by neutralization of Wnt2. The equal number of HCT116 cells were plated and treated with antibodies (IgG and TCF3 antibodies: negative controls; Wnt2 antibody). Cell proliferation was analyzed by cell counting. **J.** Rescue of Wnt nAb-induced cell growth inhibition by Wnt3A. CRC cells were treated with Wnt2 nAb and/or Wnt3A (100 ng/ml). Cell proliferation was analyzed by crystal violet staining and quantification using plate reader.

To address the pathologic role of Wnt2 in intestinal tumorigenesis, we tested the impacts of WNT2 expression to IEC proliferation. We stably transfected Wnt2 expression plasmids into CCD841CoN and assessed cell proliferation. We found that Wnt2-expressing CCD841CoN displayed the increased cell proliferation compared to empty vector transfected control cells (Figure 3A and 3B). Next, we examined the effects of *WNT2* KO on CRC cell proliferation. In contrast to the results from Wnt2 overexpression in IECs, *WNT2* KO inhibited cell proliferation of HCT116 cells (Figure 3C). Cell cycle analysis showed that *WNT2* KO HCT116 cells displayed the increase in S phase and the decrease in G0/G1 phase, compared to WT cells, as also shown in iCRT14-treated cells (Figure 3D). Similarly, Wnt2 KD also inhibits cell proliferation of CRC cell lines (HCT116, KM12, and HT15). However, HCC2998 and SW620 CRC cell lines did not show the cell growth inhibition by Wnt2 KD (Figure 3E and 3F). Given the high expression of WNT2 in CRC cells (Figure 1), we also tested whether Wnt2 induces cell proliferation in an autocrine manner. We collected conditioned medium (CM) from KM12 cells transfected with empty vector (control), shWnt2, or Wnt3A, and treated HCT116 with each CM. Then, we analyzed cell proliferation of HCT116 cells. Similar to the results from Wnt2 knockdown in CRC cells (see Figure 3A and 3B), cells treated with CM collected from Wnt2-depleted KM12 cells showed the decrease in cell proliferation (Figure 3G). However, CM from Wnt3A-overexpresing cells did not affect cell proliferation (Figure 3G). Next, we blocked the secreted Wnt2 ligands using anti-Wnt2 antibody (Wnt2 nAb). As shown in *WNT2* KO results, neutralizing Wnt2 by treatment with Wnt2 nAb downregulates *AXIN2* expression (Figure 3H) and inhibits HCT116 cell proliferation (Figure 3I). We also co-treated HCT116 cells with Wnt2 nAb and Wnt3A, a canonical Wnt ligand. We observed that Wnt2 nAb-induced cell growth inhibition was reverted by Wnt3A treatment (Figure 3J), indicating that canonical Wnt signaling mediates Wnt2-induced CRC cell proliferation. These results suggest that secreted Wnt2 is required for CRC cell proliferation.

### PRC2-mediated transcriptional repression of *WNT2*

**Figure 4:**
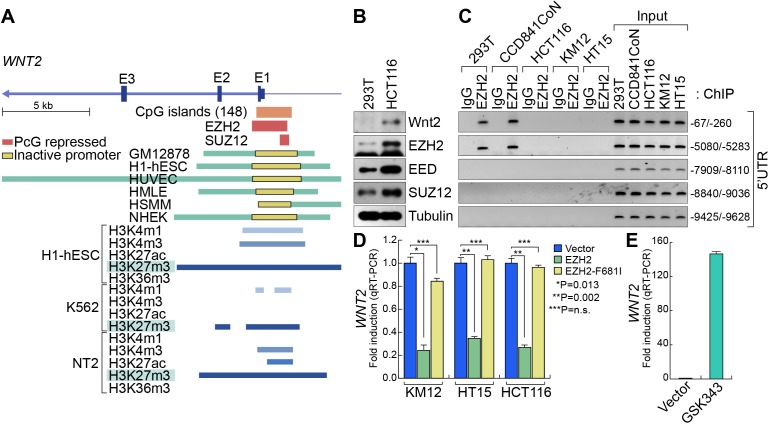
De-repression of *WNT2* in CRC **A.** *WNT2* promoter analysis. VISTA genome browser. ChIP-Seq analysis of EZH2, SUZ12, and histone modification markers in various cell lines. Of note, ChIP-Seq data show that enrichment of PRC2 components and H3K27me3 modification on *Wnt2* proximal promoter. **B.** Expression of PRC2 components. PRC2 components are highly expressed in HCT116. 293T and HCT116 cells were harvested and subjected to IB analysis for PRC2 components. Tubulin was used as an internal control. **C.** EZH2's occupancy of *WNT2* promoter. Non-CRC (293T and CCD841CoN) and CRC (HCT116, KM12, and HT15) cells were analyzed for ChIP assays. Five different regions of 5′UTR were analyzed (−67/−260; −5080/−5283; −7909/−8110; −8840/−9036; −9425/−9628). Of note, EZH2 is associated with only the proximal promoter regions (−67/−260; −5080/−5283) in non-CRC cells. **D.** Downregulation of WNT2 by ectopic expression of EZH2 in CRC. CRC cells were transfected with EZH2 (WT and F681 mutant). 48 hours after transfection, cells were analyzed for qRT-PCR. **E.** De-repression of *WNT2* by EZH2 inhibition. 293T cells were treated with GSK343 for 48 hours and analyzed for qRT-PCR.

Next, to understand how *WNT2* expression is upregulated in CRC, we analyzed *WNT2* promoter using VISTA genome browser (http://pipeline.lbl.gov/cgi-bin/gateway2). Interestingly, we found that *WNT2* proximal promoter is dominantly occupied by PRC2 (EZH2 and SUZ12) and H3K27me3 histone modification, a marker for gene repression mediated by PRC2 [44] (Figure 4A). These findings led us to test whether PRC2 represses *WNT2* transcription. Previously, it was shown that PRC2 components (EZH2, SUZ12, and EED) are highly expressed in CRC [23, 45, 46]. While PRC2 components are expressed in both 293T and HCT116 cells, the expression of EZH2, EED, and SUZ12 is slightly higher in HCT116 cells than 293T cells (Figure 4B). However, chromatin immunoprecipitation assay (ChIP) assays of *WNT2* promoter showed that EZH2 did not occupy to *WNT2* promoter in CRC cells (HCT116, KM12, and HT15), while it occupies WNT2 promoter in 293T and IECs (CCD841CoN) (Figure 4C). These results suggest that the loss of EZH2 binding to WNT2 promoter might be involved in *WNT2* de-repression in CRC cells. To test whether EZH2 is associated with transcriptional repression of *WNT2*, we ectopically expressed WT or F681I, catalytically inactive mutant, EZH2 in CRC cells (HCT116, KM12, and HT15). qRT-PCR results showed that EZH2 overexpression downregulates *WNT2* expression, whereas EZH2 F681I mutant does not (Figure 4D). Of note, the expression of Wnt3, another highly expressed Wnt ligand, was not affected by ectopic expression of EZH2 (data not shown). To complement gain-of-function approach, we also asked whether blockade of EZH2 transcriptionally activates *WNT2* in non-CRC cells. We treated 293T cells with GSK343, a specific inhibitor of EZH2 [47]. We found that GSK343 treatment induces the significant de-repression (upregulation) of *WNT2* expression in 293T cells (Figure 4E). These results suggest that *WNT2* expression is transcriptionally repressed by PRC2 in non-CRC cells and de-repressed by the loss of PRC2's promoter occupancy in CRC cells.

## DISCUSSION

**Figure 5:**
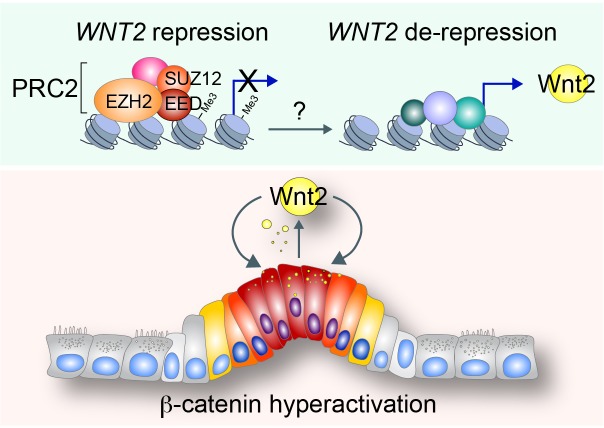
Illustration of working model In normal intestine, *WNT2* expression is repressed by PRC2-induced H3K27me3 histone modification of *WNT2* proximal promoter. By unknown mechanisms, PRC2's repressive function on *WNT2* expression is inhibited and *WNT2* expression is de-repressed. Subsequently, Wnt2 activates canonical Wnt signaling during intestinal tumorigenesis, which leads to maintenance of hyperactivation of Wnt/β-catenin signaling for CRC cell proliferation.

In this study, we found that Wnt2 is highly upregulated in CRC and complements Wnt/β-catenin signaling for CRC cell proliferation (Figure 5). Despite homogeneous genetic mutations in *APC*, *CTNNB1,* or *AXIN2*, CRC cells show heterogeneous nuclear localization of β-catenin [48, 49], which is called ‘β-catenin paradox’. Accumulating evidences have supported this model: (a) Rather than having an absolutely higher level of β-catenin protein, fold-induction of β-catenin is critical for β-catenin target gene activation [50]. (b) Mutant APC protein partially functions in protein destruction complex to target β-catenin protein [14]. (c) Blockade of Wnt ligands induces growth inhibition and apoptosis in cells harboring *APC* mutation [14, 51], so does secreted frizzled-related proteins (SFRPs) (secreted Wnt antagonists)[48, 52]. (d) Genetic mutation in E3 ligases (RNF43 and ZNRF3) targeting Wnt receptors contributes to CRC [53, 54]. (e) Moreover, Tankyrase inhibitor (blocking Axin1 degradation for subsequent β-catenin inhibition) suppresses CRC cell proliferation [55]. (f) It was shown that Ras/MAPK signaling is required for Wnt/β-catenin signaling activation [56, 57]. Therefore, we hypothesized that additional stimuli at the upstream of protein destruction complex (APC, GSK3, Axin, and CK1) complement Wnt/β-catenin signaling activity in CRC. Among 19 Wnt ligands, we found that Wnt2 is most significantly upregulated in CRC, which led us to investigate the potential tumorigenic roles of Wnt2 in CRC.

We initially questioned whether hyperactivated β-catenin by genetic mutations is further activated by additional Wnt signaling stimuli. To address this, we examined whether GSK3 inhibition stabilizes (activates) β-catenin protein. We inhibited GSK3, a component of protein destruction complex targeting β-catenin for ubiquitin-mediated protein degradation, using LiCl. Interestingly, LiCl treatment not only upregulates β-catenin protein but also activates β-catenin reporter activity in CRC cells despite genetic mutations in *CTNNB1*/β-catenin or *APC* of CRC cells (Supplementary Figure 2). Interestingly, HCT116 and KM12 cells show the significant decrease in cell proliferation by depletion of Wnt2 (Figure 3E and 3F), which correlates with LiCl-induced stabilization of β-catenin protein (Supplementary Figure 2). Of note is that SW620 and HCC2998 CRC cells did not exhibit cell growth inhibition by Wnt2 depletion (Figure 3F), which is also consistent with the results that LiCl does not stabilize beta-catenin protein and not activate beta-catenin reporter activity (Supplementary Figure 2). Considering the high expression of Wnt2 in all CRC cells (Figure 1D), it is plausible that other Wnt ligands such as Wnt3 might compensate the loss of Wnt2 in SW620 or HCC2998 cells. Thus, comprehensive analysis of highly expressed Wnt ligands (Wnt2, Wnt3, Wnt5A, and Wnt7b) is necessary. Nonetheless, these results imply that additional stimuli can further activate Wnt signaling in CRC cells. In regard to this, our finding strongly suggests that Wnt2 is a crucial factor for maintaining Wnt signaling activation in CRC, in addition to genetic activation of Wnt signaling. It is noteworthy that *APC* mutations are mainly observed in CRC cells regardless of mutations or aberrant expression of *AXIN2*, *CTNNB1* (Supplementary Figure 3), which indicates the insufficiency of *APC* mutation per se in fully competent Wnt signaling hyperactivation during intestinal tumorigenesis. In line with this, Voloshanenko et al. recently showed that *APC* mutation is not sufficient to completely activate Wnt signaling in CRC cells. They proposed that Wnt3A is required for complete activation of Wnt signaling [14]. However, our in silico analysis indicates that *WNT2*, *WNT5A*, and *WNT3*, but not *WNT3A* are significantly upregulated in CRC (Figure 1A and 1B). Moreover, depletion of endogenous Wnt2 downregulates Wnt signaling activity and inhibits CRC cell proliferation (Figures 2 and 3). Thus, Wnt2 is more likely to be associated with the maintenance of Wnt signaling activity in CRC. To better address this, it is necessary to directly compare the impacts of Wnt2 and Wnt3A to Wnt signaling activation and CRC cell proliferation. Additionally, *K-Ras* mutation was shown to be required for activation of β-catenin in CRC [57]. However, CRC cell lines with mutations in either *K-Ras* or *BRAF* (Supplementary Figure 4) also exhibit Wnt2 dependency on cell proliferation and β-catenin activation, which suggests that the impact of Wnt2 to β-catenin stabilization might be independent of Ras/MAPK signaling.

We found that EZH2 regulates transcriptional repression of *WNT2*. EZH2, a core component of PRC2, silences target gene transcription by histone modification (H3K27me3). EZH2 and SUZ12 specifically occupy the proximal promoter of *WNT2* and induce H3K27me3, which leads to transcriptional inactivation of WNT2 in non-CRC cells. However, CRC cells exhibit the loss of EZH2 association with *WNT2* promoter, which leads to de-repression of *WNT2* expression. Due to the expression of PRC2 components (EZH2, EED, and SUZ12) in both non-CRC and CRC cells (Figure 4B), it is plausible that EZH2's activity or target gene selection might be specifically modulated by additional factors. For example, posttranslational modification of EZH2 was shown to modulate EZH2-mediated histone modification [58, 59], which might facilitate release of EZH2 from *WNT2* promoter and induces de-repression of *WNT2* transcription. Another possible explanation of regulatory mechanism of EZH2 is the sequestration of EZH2 from PRC2 to β-catenin transcriptional complex. Previously, we found that PAF (PCNA-associated factor)/KIAA0101 is specifically expressed in CRC cells but not in normal IECs. PAF binds to β-catenin and sequesters EZH2 from PRC2 to β-catenin transcriptional complex, resulting in transcriptional activation of β-catenin target genes in CRC [42]. Thus, it is highly likely that PRC2-mediated repression of *WNT2* is diminished by the loss of EZH2 in PRC2, which subsequently leads to de-repression of *WNT2* in CRC. This should be addressed in future studies. Although the expression of PRC2 components is elevated in CRC, it is noteworthy that *WNT2* expression is mutually exclusive with the expression of EZH2, EED, and SUZ12 (Supplementary Figure 5). These in silico results support our model that PRC2 mediates repression and de-repression of WNT2 in normal cells and CRC cells, respectively. Importantly, the multiple Kaplan-Meier survival analyses suggest that high expression of EZH2 is associated with relapse-free survival [46], indicating that high EZH2 expression correlates with improved survival of CRC patients. These pathologic results also support our finding that EZH2 negatively regulates the expression of *WNT2*, another key factor for CRC cell proliferation.

Taken together, our findings suggest that Wnt2 plays a critical role in complementing Wnt signaling in CRC. Furthermore, our study proposes that rather than blockage of core-components of Wnt signaling, molecular intervention of Wnt2 might be beneficial to translation into CRC therapy, by minimizing normal tissue damages.

## MATERIALS AND METHODS

### Oncomine database analysis

cDNA microarray datasets of colon adenocarcinoma and normal tissue samples were analyzed using Oncomine database (www.oncomine.org). P < 0.0001; fold change >2; 10% top ranked.

### Immunohistochemistry

Human CRC tissue microarray was purchased from Biomax (Co1002), and immunostained with anti-Wnt2 antibody (SantaCruz), as previously performed [60]. For immunostaining of tumor samples from xenograft transplantation, samples were collected and fixed with 10% formalin. After processing for paraffin embedding, sectioned samples were immunostained followed by standard protocols.

### Mammalian cell culture and constructs

All cell lines were purchased from American Type Culture Collection (ATCC) and maintained in Dulbecco's modified Eagle medium (containing 10% fetal bovine serum and 1% penicillin-streptomycin). For gene depletion, shRNA lentiviruses (shGFP or shWnt2) (Sigma; MISSION shRNA) were stably transduced into target cells using puromycin selection (1 to 2 μg/ml). Wnt2 expressing plasmid was purchased from Addgene. GSK343 was purchased from Sigma.

### Immunoblotting

Proteins were obtained as previously described [42], and the following antibodies were used for Immunoblotting: β-catenin (Cell Signaling), active β-catenin (Millipore), Tubulin (Sigma), FLAG (M2; Sigma), Wnt2 (SantaCruz), Wnt3a (Cell Signaling), Wnt5a (Cell Signaling), pLRP6 (Cell signaling), LRP6 (Cell signaling), LRP5 (Cell signaling), Dvl2 (Cell signaling), Dvl3 (Cell signaling), Axin1 (Cell signaling) and GSK3β (BD Bioscience), EZH2 (Cell signaling), EED (Millipore), and SUZ12 (Abcam).

### Reporter assays

The reporter plasmids, pMegaTOPFLASH and pMegaFOPFLASH, were transiently transfected with pSV40-Renilla and analyzed using Dual luciferase assay system (Promega), as previously performed [42].

### WNT2 somatic gene targeting

The KO cells were generated by CRISPR using a lentiviral vector [61]. The lentiviral plasmid contains two expression cassettes, hSpCas9 and the chimeric gRNA. gRNAs were designed based on the protospacer adjacent motif (PAM) on the target site, using standard cloning methodology generating lentiCRISPR. The lentiCRISPR plasmids were transfected into HEK293T cells along with packaging plasmids pCMV-ΔR8.2 dvpr and pCMV-VSVG for lentiviral packaging. CRC cell lines were then transduced with the lentivirus and selected in puromycin for 72 hours. The KO was confirmed by immunoblotting and genomic DNA sequencing. gRNA sequences: #1: 5′- CCATG AAGAG TTGAC CTCGG-3′; #2: 5′- ACCAT GAAGA GTTGA CCTCG-3′.

### Gene expression analysis

RNAs were extracted by TRIzol (Invitrogen) and converted into cDNAs using SuperScript II (Invitrogen) with random hexamer. For gene expression analysis, qRT-PCR was performed. qRT-PCR results were quantified by comparative 2^−ΔΔCt^ methods. For internal controls, *HPRT* was used. qRT-PCR primer sequence information: *Wnt2* forward/reverse: 5′CCC ACA GCA CAT GAC TTC AC3′/5′CTG TAT CAG GGA CCG AGA GG3′; *Axin2* forward/reverse: 5′ CTC CTT GGA GGC AAG AGC3′/5′ GGC CAC GCA GCA CCG CTG3′; *HPRT* forward/reverse: 5′GCT ATA AAT TCT TTG CTG ACC TGC TG3′/5′AAT TAC TTT TAT GTC CCC TGT TGA CTG G3′

### Cell proliferation analysis

For FACS analysis, cells were fixed with 70% ethanol and stained with propidium iodide, then subjected to cell cycle analysis using FACSCalibur (Becton Dickinson FACSCalibur), as previously performed [62]. For cell proliferation analysis, fourteen days after seeding the cells, we fixed the cells with 10% formalin and stained them with crystal violet for 30 min. For quantitative analysis, cells stained with crystal violet were subjected to lysis with 1% sodium dodecyl sulfate (SDS), and absorbance was measured at 590 nm using a 96-well microplate reader (BioTek microplate reader).

### ChIP assay

ChIP assays were performed as previously performed [42]. The antibody against EZH2 was purchased from cell signaling. ChIP primer sequence information:

−9425/−9628 forward/reverse: 5′ctctt gaatc tgggc aggtc3′/5′gaaag attga ctggg ctgga3′; −8840/−9036 forward/reverse: 5′cgtta atccc accac tttgg3′/5′tacaa cctct gcctc cttgg3′; −7909/−8110 forward/reverse: 5′ttttg ccttt cacca aaacc3′/5′tagtg agtgg ccagg agacc3′; −5080/−5283 forward/reverse: 5′ttcag gtttt tggcg tctct3′/5′atgcg cctgt gtgta tgtgt3′; −67/−260 Forward/reverse: 5′tgctt tggca gatac tgctg3′/5′ctgaa gctgg gatga agagc3′

### Statistical analysis

The Student's t-test was used for comparisons of two groups (n ≥ 3). P values less than 0.05 were considered significant. Error bars indicate standard deviation.

## SUPPLEMENTARY FIGURES AND TABLES


