# Supplementary material for: Wnt2 complements Wnt/β-catenin signaling in colorectal cancer
Source: Oncotarget. 2015 Oct 15;6(35):37257–68. doi: 10.18632/oncotarget.6133 (PMC4741928; doi:10.18632/oncotarget.6133)
Supplement: Supplementary file 1 [file oncotarget-06-37257-s001.pdf]

Wnt2 complements Wnt/β-catenin signaling in colorectal cancer

Supplementary Material

Rank	P-value	Fold Change	Gene	Reporter
18	2.05E-32	19.29	WNT2	A_23_P59806
54	4.53E-28	5.63	AXIN2	A_23_P148014
806	3.04E-16	2.78	WNT5A	NM_003392_2_4162
1755	6.06E-12	3.28	WNT3	A_23_P130153
1796	8.31E-12	1.71	WNT11	A_24_P35640
3286	6.79E-8	2.10	WNT7B	A_23_P320054
5146	4.65E-5	1.25	WNT3A	NM_033131_2_2826
6464	9.44E-4	1.27	WNT10B	A_23_P162321
8731	0.030	1.14	WNT9B	A_23_P89588
8870	0.034	1.21	WNT8A	NM_058244_1_1830
9001	0.039	1.09	WNT7A	NM_004625_2_1449
9494	0.067	1.14	WNT8B	NM_003393_2_1294
10211	0.127	1.24	WNT6	A_24_P208513
13799	0.778	-1.08	WNT16	NM_057168_1_3060
13874	0.791	-1.09	WNT4	NM_030761_2_303
15449	0.977	-1.20	WNT9A	A_23_P160680
15704	0.986	-1.37	WNT10A	A_23_P102113
15901	0.991	-1.15	WNT1	NM_005430_2_2162
18978	1.000	-1.84	WNT5B	A_23_P53588
20194	1.000	-3.13	WNT2B	A_23_P138347

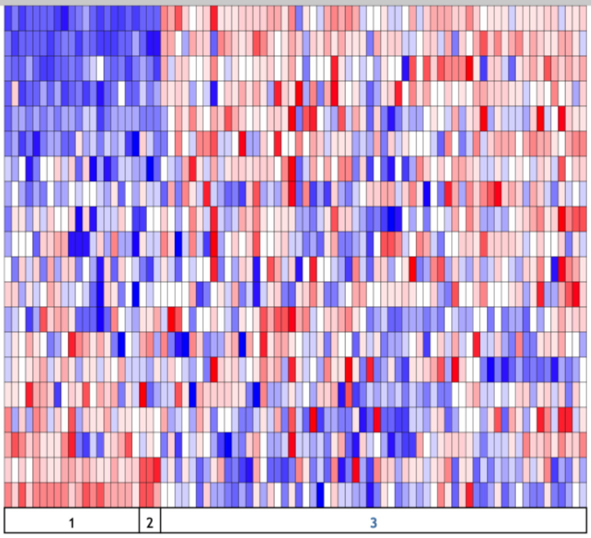

Supplementary Figure 1. Correlation between Wnt ligands and *AXIN2* expression

Oncomine analysis of TCGA dataset (TCGA Colorectal, 237 samples, 20,423 measured genes); colon (19), rectum (3), and rectal adenocarcinoma (60); downregulation (blue), upregulation (red). *WNT2* (the first row; 19.29 fold increase) and *AXIN2* (the second row; 5.63 fold increase) display the highest expression in CRC cells, with the significantly low P values (2.05E-32 and 4.53E-28, respectively).

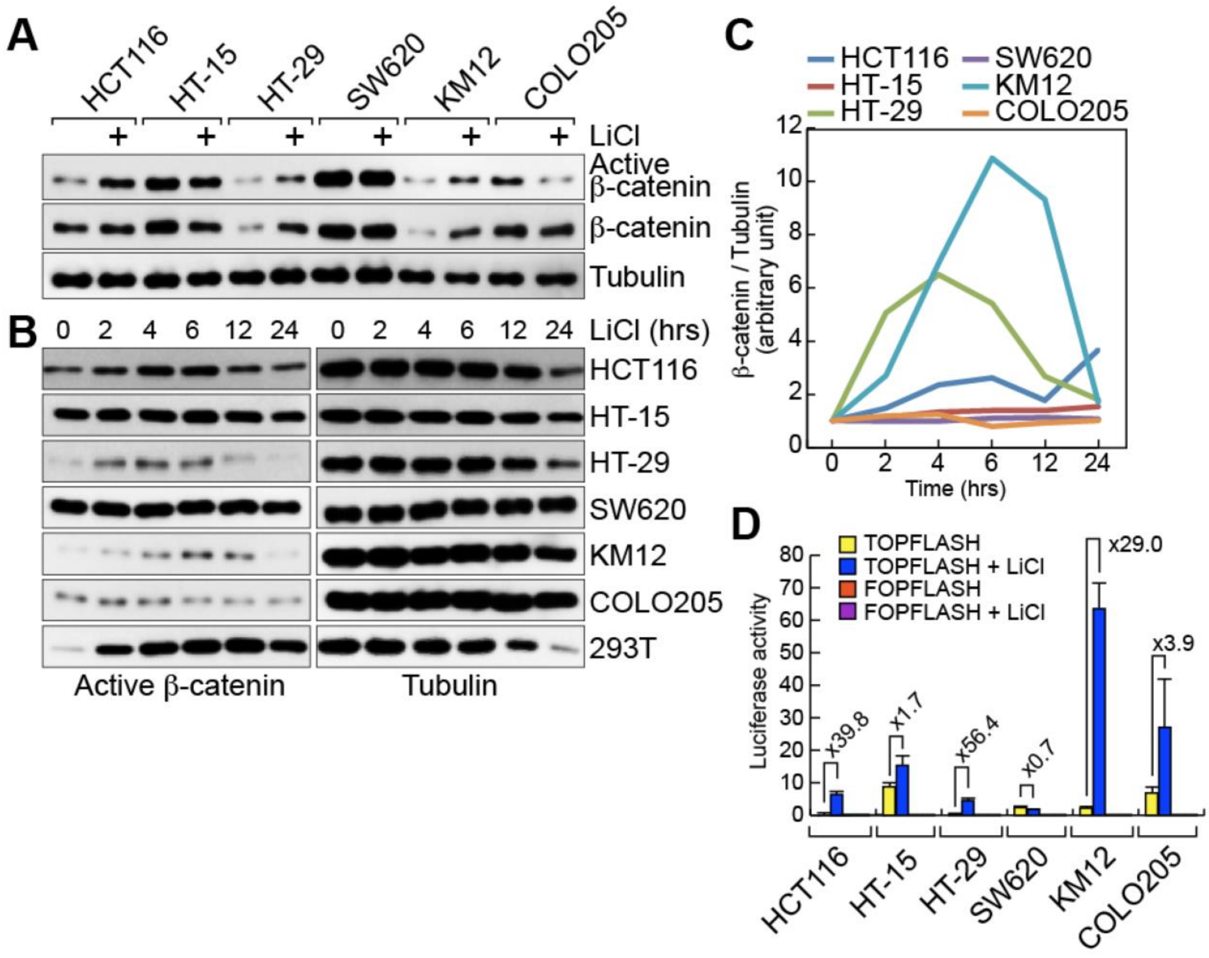

Supplementary Figure 2. Stabilization of β-catenin by GSK3 inhibition in CRC cells

(A~C) Stabilization of β-catenin by GSK3 inhibition in CRC cells. CRC cell lines were treated with LiCl (25 mM, 4 hours) and subjected to IB assays. LiCl treatment increases the level of active β-catenin as well as total β-catenin (A), also in a time-dependent manner (B). Kinetics of β-catenin protein stabilization by LiCl was plotted. Images were quantified using ImageJ (C). (D) β-catenin reporter activation by LiCl in CRC cells. CRC

cells were transfected with β-catenin reporter plasmids (TOPFLASH or FOPFLASH). 24 hours after transfection, cells were treated with LiCl (25 mM, 4 hours). 24 hours after LiCl, cells were collected for luciferase assays.

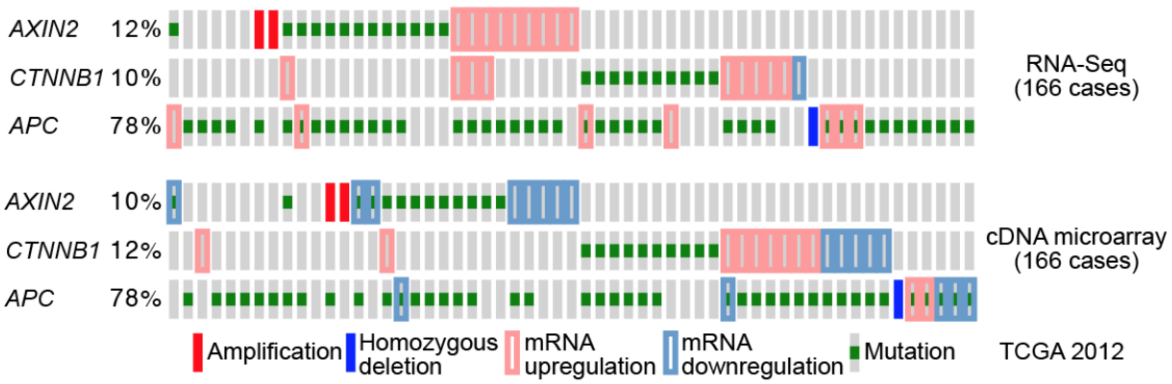

Supplementary Figure 3. Mutually exclusive gene alteration among Wnt signaling components

cBioPortal analysis of TCGA 2012 colorectal carcinoma data sets. 166 patient/case sets. Genome profiles: mutation, copy number alterations from GISTIC, and mRNA expression x-Scores (RNA-Seq V2 RSEM). It is noteworthy that CRC patient samples harboring genetic mutations or mRNA upregulation of *CTNNB1* or *AXIN2* also contain *APC* mutation.

Cell line	*APC*	*CTNNB1*	*AXIN1*	*BRAF*	*PTEN*	*K-Ras*	*p53*
HCT116	WT	∆S45	WT	WT	WT	G13D	WT
HT-29	E853X	WT	WT	V600E	WT	WT	R273H
HCT-15	I1417fs	WT	WT	WT	WT	G13D	S241F
SW620	Q1338X	WT	WT	WT	WT	G12V	R273H, P309S
KM12	N1818fs	WT	E640fs (Del)	WT	G129X	WT	H179R
COLO205	E1554fs	WT	-	V600E	WT	WT	G266E

Supplementary Figure 4. Genetic mutations in CRC cell lines

WT: wild-type; Del: deletion; fs: frame-shift; X: stop codon (termination).

Gene	*JARID2*	*AEBP2*	*EZH2*	*EED*	*SUZ12*	***Wnt2***	*RBBP7*	*RBBP4*
JARID2	-	0.000834	0.12952	0.718848	0.643482	0.574192	0.369384	0.00719
AEBP2		-	0.354056	0.65317	0.000671	0.594107	0.648737	0.842242
EZH2			-	0.053337	0.595414	0.614593	0.048378	0.148295
EED				-	0.140013	0.679618	0.274565	0.119588
SUZ12					-	0.702526	0.254302	0.890087
Wnt2						-	0.853921	0.939255
RBBP7							-	0.949247
RBBP4								-

p values are indicated in each cell

Strong tendency towards mutual exclusivity (0 < odds ratio < 0.1)

No association (0.5 < odds ratio < 2)

Tendency toward co-occurence (2 < odds ratio < 10)

Strong tendency toward co-occurence (odds ratio > 10)

www.cbioportal.org
TCGA 2012, colorecal adenocarcinoma
Patient/case set: Tumor CNA (195)
Genome profiles:
mutations
copy number alterations from GISTIC
mRNA expression z-Scores (RNA-Seq V2 RSEM)

Supplementary Figure 5. Mutually exclusive gene alteration between PRC2 and *WNT2*

cBioPortal analysis of TCGA 2012 colorectal carcinoma data sets. 195 patient/case sets. Genome profiles: mutation, copy number alterations from GISTIC, and mRNA expression x-Scores (RNA-Seq V2 RSEM). P values are indicated in each cell. Red: strong tendency toward mutual exclusivity; empty: no association; yellow: tendency toward co-occurrence; green: strong tendency toward co-occurrence.
